# Restricting C-Reactive Protein Use in Early-Onset Neonatal Sepsis Reduces Unnecessary Antibiotic Exposure

**DOI:** 10.3390/antibiotics15030308

**Published:** 2026-03-18

**Authors:** Valeria Capone, Sophie Venturelli, Eleonora Cresta, Francesca Miselli, Martina Buttera, Licia Lugli, Eugenio Spaggiari, Alberto Berardi

**Affiliations:** 1 Pediatric Post-Graduate School, University of Modena e Reggio Emilia, 41125 Modena, Italy; 2 Department of Maternal and Child Health, School of Pediatrics Residency, Policlinico Umberto I, Sapienza University of Rome, 00185 Rome, Italy; 3 Neonatal Intensive Care Unit, Department of Maternal and Child Health, University Hospital of Modena, 41124 Modena, Italy; 4 PhD Program in Clinical and Experimental Medicine, University of Modena and Reggio Emilia, Via Università, 41121 Modena, Italy

**Keywords:** early-onset sepsis, newborn, antimicrobial stewardship

## Abstract

**Background:** some consensus guidelines include C-reactive protein (CRP) in the diagnostic workup of early-onset neonatal sepsis (EOS), but its routine use remains debated due to variable diagnostic performance. The experiences and data from individual centers can help clarify its clinical utility and inform local practice. **Methods:** Retrospective analysis at a level III center assessing the impact of discontinuing routine C-reactive protein (CRP) testing for suspected early-onset sepsis (EOS). Laboratory use, antibiotic therapy, and outcomes in neonates of all gestational ages were compared before (2021–2022) and after (2024–2025) the policy change. **Results:** A total of 638 neonates were included (period 1, *n* = 348; period 2, *n* = 290). CRP testing decreased markedly (218/348 in period 1 vs. 40/290 in period 2; *p* < 0.001), alongside a significant reduction in the number of complete blood counts performed (285/348 vs. 214/290; *p* = 0.02). Concurrently, both the proportion of short antibiotic courses (≤48 h) initiated within the first 3 days of life (98/181 vs. 88/133) and the median duration of antibiotic therapy (48.0 h vs. 40.0 h; *p* < 0.001) decreased without worsening outcomes. The duration of antibiotic therapy was even shorter in infants born before 34 weeks’ gestation (48.0 h vs. 37.5 h; *p* < 0.001). **Conclusions:** Restricting the use of CRP in the evaluation of EOS was associated with a reduction in unnecessary antibiotic exposure. This strategy may be considered a core component of neonatal antibiotic stewardship programs.

## 1. Introduction

Early-onset neonatal sepsis (EOS) is typically defined by the onset of symptoms within 72 h of life and by confirmation of a pathogen in normally sterile body fluids (such as blood or cerebrospinal fluid) [1]. Group B *Streptococcus* and *E. coli* are the most frequently responsible pathogens and account for about 70% of cases [2,3]. EOS is still associated with significant mortality and risks of long-term sequelae, especially in preterm infants. However, the clinical presentation may be poorly specific, and at present reliable early markers are lacking, while risk factors have insufficient predictive value [4].

Antimicrobials are therefore the drugs most prescribed by neonatologists. Approximately 14% of late preterm and term neonates and up to 90% of extremely low birth weight infants receive empirical antibiotics immediately after birth, although culture-proven EOS occurs in only a minority of these infants [5,6]. However, exposure to antibiotics in the first days of life may have important adverse effects. It may increase the risk of antimicrobial resistance and lead to dysbiosis, with a potential negative long-term impact (diabetes, obesity, asthma, inflammatory bowel disease, neurodevelopmental disorders, atopic dermatitis, and multiple sclerosis) [7,8,9,10].

C-reactive protein (CRP) is an inflammatory biomarker frequently used as a diagnostic aid to confirm or rule out sepsis, ideally by providing additional information beyond risk-based history and clinical status [11,12]. However, recent studies have highlighted the ongoing variability and uncertainty surrounding CRP use in EOS evaluations [13,14].

CRP is commonly used to assess the inflammatory response associated with sepsis, based on the theoretical assumption that neonates with EOS may have sterile blood cultures due to limited culture sensitivity, particularly following maternal intrapartum antibiotic prophylaxis (IAP) [15]. Accordingly, CRP is often measured early, at the time empiric antibiotics are initiated for suspected EOS, even though the sensitivity of CRP is lowest during the early stages of infection [16]. CRP is also used to support decisions to discontinue antibiotics early (to rule out EOS), as endorsed by the NICE guidelines [17], based on the high negative predictive value of repeated normal CRP measurements [18]. However, recent studies have failed to demonstrate that this approach effectively reduces the overall duration of antimicrobial therapy [13,14]. Finally, when cultures remain sterile but EOS is still clinically suspected, clinicians often rely on CRP trends to guide antibiotic management. However, elevated CRP in neonates more often reflects non-infectious inflammation—such as transient tachypnea, respiratory distress, meconium aspiration, fetal distress, maternal fever, or prolonged membrane rupture—rather than EOS. [18,19,20,21]

Some diagnostic algorithms [22] and guidelines [17,23,24] include CRP—alone or combined with procalcitonin—in the initial workup, while others do not [25,26]. Insufficient information on CRP use is available among Italian centers.

Here, we report retrospective data from a level III center in which CRP testing was removed from the routine diagnostic work-up for EOS at all gestational ages. We compared CRP and CBC testing volume, and the frequency and duration of antibiotic use as well as cases of culture-proven EOS and their clinical outcomes, before and after implementation of the new approach, when CRP was no longer routinely measured in every case of suspected sepsis.

## 2. Results

**Figure 1:**
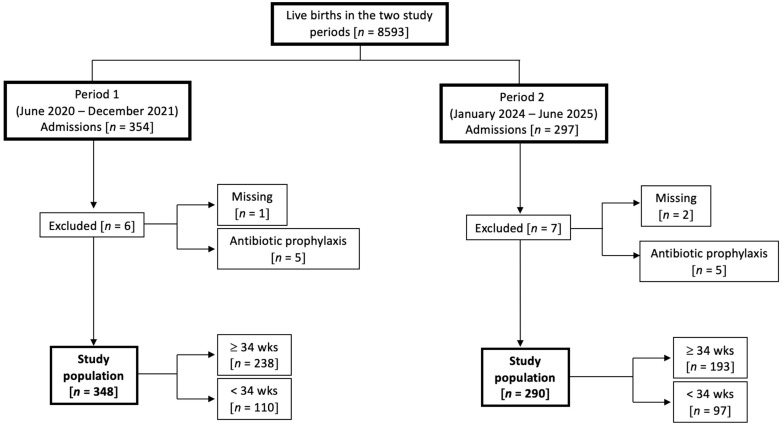
Flow chart of the newborn selection.

During the two study periods there were 651 neonates admitted to NICU within 72 h of birth, of which 13 were excluded, leaving 638 neonates for the final analysis (period 1, *n* = 348; period 2, *n* = 290). Figure 1 displays the study population selection according to their gestational age. Rates of transient tachypnea of the newborn, meconium aspiration syndrome or septic shock were 7.9%, 3.2%, and <1% respectively. Case fatalities (from 0 to 30 days of life) due to all causes were 11 in period 1 and nine in period 2. Fatalities attributable to confirmed EOS included two cases in period 1 (one caused by *E. coli* and one by CoNS) and one case in period 2 (caused by CoNS). Table 1 shows demographics of the study population. During period 2, neonates were more likely to be delivered after maternal intrapartum fever.

**Figure 2:**
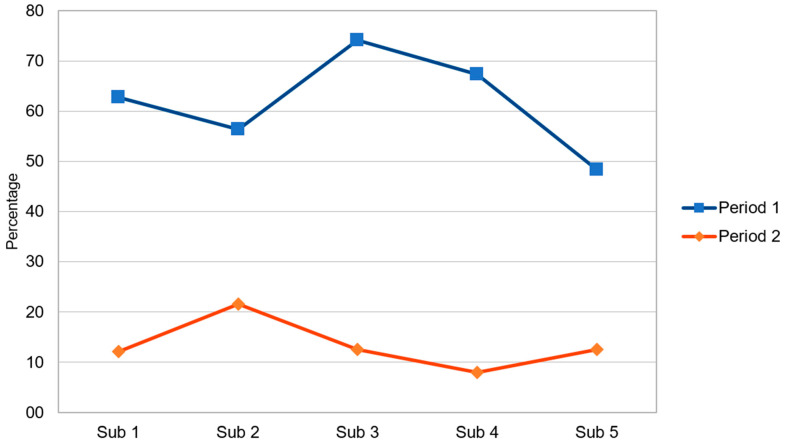
C-reactive protein measurements. Period 1 and period 2 are segmented into five intervals of roughly four months.

Comparisons (period 1 vs. period 2) of laboratory evaluations among uninfected neonates treated with antibiotics from 0 to 3 days of life are reported in Table 2. Blood cultures obtained and CRP testing were markedly reduced, along with a reduction in the number of complete blood counts performed. Figure 2 displays CRP measurements in periods 1 and 2, segmented into five intervals of roughly four months.

The proportion of neonates who were never exposed to antibiotic treatment (within the first 15 days of life or during their entire hospital stay, if shorter) was 154/348 (44.3%) in Period 1 and 146/290 (50.3%) in Period 2, with no evidence of a statistically significant difference between the two periods (*p* = 0.15).

**Figure 3:**
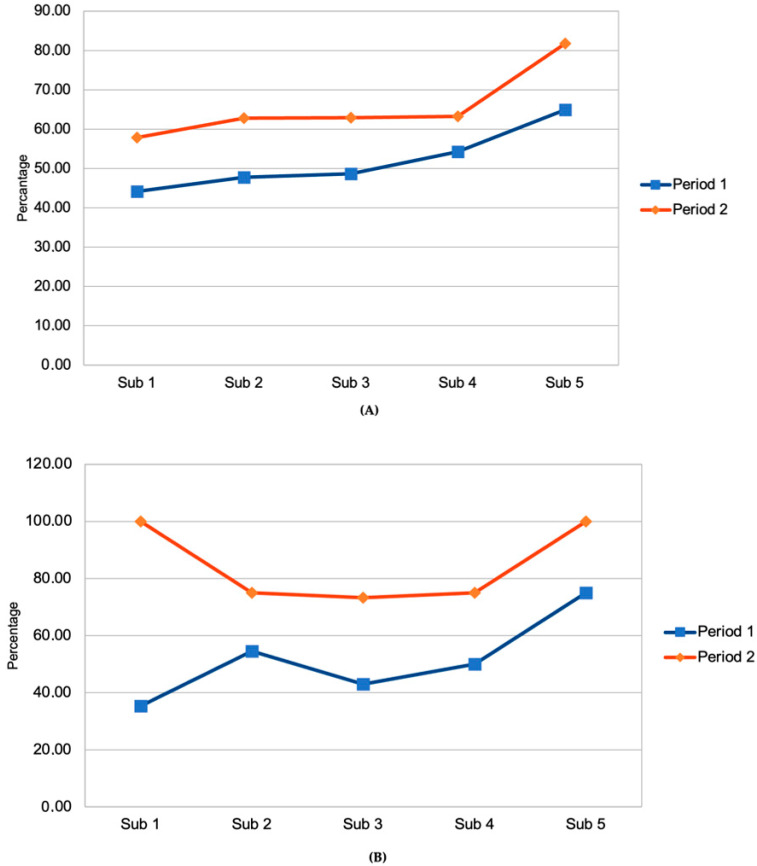
Short antibiotic courses. Periods 1 and 2 are segmented into five intervals of roughly four months. All neonates (**A**) and preterm neonates under 34 weeks’ gestation (**B**) are displayed separately.

Table 3 displays antibiotic exposure in all neonates and neonates under 34 weeks’ gestation. Short antibiotic courses (≤48 h), median hours of duration, and DOT were significantly reduced in Period 2. The median hours of duration were further reduced among neonates under 34 weeks’ gestation. Figure 3 displays short antibiotic courses administered in periods 1 and 2, segmented into five intervals of roughly four months in all neonates (A) and preterm neonates under 34 weeks’ gestation (B). In both periods, there appears to be an increasing trend in the use of short courses of empirical antibiotic therapy.

Among infants treated with a short antibiotic course, antibiotics were re-initiated within 7 days after discontinuation in 5/98 infants in period 1 and in 4/88 infants in period 2 (*p* = 0.85).

The number of neonates who underwent repeat blood cultures from 72 to 168 h of life for suspected sepsis (after receiving an antibiotic treatment initiated during the first 72 h of life) was 18/181 (9.9%) in period 1 and 18/133 (13.5%) in period 2 (*p* = 0.42).

We calculated the length of hospital stay after excluding all preterm neonates, as hospitalization duration in this group may be strongly influenced by gestational age. Among term neonates, no significant differences in length of stay were observed between the two periods (Period 1: median 6 days, IQR 4.0–11.0 vs. Period 2: median 5 days, IQR 3.0–10.0; *p* = 0.126).

## 3. Discussion

Centers that have de-emphasized routine CRP testing in their EOS evaluations have demonstrated reductions in antibiotic use without worsening neonatal outcomes [13,27,28].

We did not rely on procalcitonin (PCT) for the diagnosis of EOS, as reference ranges during the first 72 h of life remain insufficiently established. Moreover, similar to CRP, PCT concentrations may be influenced by a variety of non-infectious conditions. The limited specificity and suboptimal positive predictive value of PCT, as well as other currently available biomarkers, may consequently contribute to the administration of antibiotic therapy in newborns who are not infected [4,11,18,29,30].

We evaluated the impact of reducing the use of CRP on antibiotic exposure and diagnostic workup of EOS. Benchmarking one’s own NICU against studies from other settings and institutions can stimulate critical appraisal of local practices and foster re-evaluation of established clinical approaches. Because this policy change was implemented in a clinical setting with long-standing and well-established antimicrobial stewardship practices, [31,32] confounding from concurrent changes in sepsis management was probably minimized, revealing the key role of CRP restriction on antibiotic use.

Although WBC testing declined in period 2 alongside CRP, the reduction was modest (from 82% to 74% of neonates), whereas CRP testing dropped sharply—by more than fourfold. These findings suggest that reduced CRP testing was a key driver of the decrease in empiric antibiotic therapy. Blood culture collection also decreased; however, in period 2, the number of cultures remained substantially higher than the number of neonates treated with antibiotics, effectively ruling out a systematic omission of cultures in potentially infected infants.

The proportion of neonates undergoing lumbar puncture was low and did not differ substantially from the ~15% reported in a recent U.S. study involving two NICUs [30]. In the era of IAP, early-onset meningitis has become a less common manifestation of EOS, [33,34,35] and some clinicians may therefore be more reluctant to perform lumbar puncture to rule out meningitis, especially when neonates are only mildly ill. Although our low proportion might raise concerns about potential underestimation of meningitis cases, the vast majority of neonates in our cohort received very short antibiotic courses, making it unlikely that meningitis was underestimated.

Reliance on CRP to guide neonatal management, as recommended in some guidelines, [17] has been associated with higher rates of lumbar puncture among uninfected neonates [36] and increased length of hospital stay [36,37]. However, in contrast to these studies, we did not confirm shorter lengths of stay.

**Our most clinically meaningful finding was a substantial reduction in antibiotic use during the first days of life among uninfected neonates**. In period 2, uninfected infants were more likely to receive short courses (≤48 h), and overall antibiotic exposure decreased further. The duration of antibiotic therapy, measured in hours, decreased by approximately 17% overall, with an even larger reduction (~22%) in infants born before 34 weeks’ gestation, who are at highest risk of prolonged exposure [38]. Sepsis-related mortality and adverse outcomes remained unchanged.

This study has important limitations, mainly due to its retrospective design, which limits the ability to attribute the observed changes to a single factor (CRP). For example, the reduction in blood culture and CBC rates, together with a gradual increase in short-course antibiotic treatments, suggests that the intervention consisted of a coordinated set of changes; multiple factors—including new algorithms, staff education, changes in the antibiotic threshold, and temporal trends—may have contributed to the observed effects. However, to minimize the impact of potential confounding, we compared two closely spaced time periods. The changes were implemented concurrently in both the NICU and the intermediate care ward; since the same clinicians rotated between the two units, the entire staff followed consistent practices.

Finally, in period 2 more infants were born to mothers with intrapartum fever, which could affect results. However, data on maternal fever were partially incomplete in period 1; furthermore, postpartum antibiotic use would be expected to increase (rather than decrease), since the risk profile was higher in period 2.

## 4. Materials and Methods

### 4.1. Study Design

This observational, retrospective study was carried out in the neonatal intensive care unit (NICU) of the University Hospital of Modena, Italy; this is a high-volume level three facility, with inborn neonates accounting for most admissions. The NICU contains 20 cots, receives approximately 400 admissions per year, and the medical staff consists of 12 physicians. The study project was approved by the local ethics committee (Protocol AOU 0002163/2024, subsequently amended).

The study concerns all neonates admitted to the NICU (code 0.73 for Italy) during two periods: (i) baseline (when CRP was included in the diagnostic panel of suspected EOS), from 1 June 2020 to 30 December 2021 (live births *n* = 4319) and (ii) intervention (after removing the routine CRP measurement from the diagnostic panel of suspected EOS in the first week of life), from 1 January 2024 to 30 June 2025 (live births *n* = 4274). During period 2, CRP was suggested only in the case of culture-proven or focal infection (meningitis, necrotizing enterocolitis or pneumonia). We selected a relatively short interval between the two periods to better isolate the impact of changes in CRP measurements, while ensuring that newborns were otherwise managed in a largely comparable manner. However, between these two periods, procedures were put in place to inform the medical and nursing staff of the NICU that diagnostic approach was changed. Algorithms for guiding the use of antibiotics were created and the empirical use of antimicrobials was revised: broad-spectrum antibiotics for EOS (ampicillin plus gentamicin) were discontinued within 36–48 h (to rule out sepsis) based on: (i) negative blood and cerebrospinal fluid culture, (ii) absence of focal infection (pneumonia, meningitis), and (iii) clinical improvement of the patient. A treatment duration of ≥5 days was recommended for pneumonia or suspected (culture negative) sepsis [31,39,40]. Third generation cephalosporins were administered for suspected meningitis pending culture results. After pathogen isolation, broad- were replaced by narrow spectrum antibiotics, based on known antimicrobial susceptibility [7,39].

The primary outcome measure of the study was to assess changes in the frequency of complete blood count and C-reactive protein (CRP) testing, and to determine whether antibiotic use differed between the baseline and the intervention period. As a secondary outcome, we assessed whether there was any clinical worsening due to the change in antibiotic use strategy. The number of fatal cases related to culture-proven or suspected sepsis as well as the number of repeated blood cultures after discontinuing antibiotics were compared between period 1 and period 2.

### 4.2. Clinical Management of Infants

In our NICU, the serial clinical examination approach (neonates at ≥34 weeks’ gestation) is applied to asymptomatic infants at risk, who undergo a structured program of standardized serial clinical assessments over the first 48 h of life [41]. After implementing this SCO approach, antibiotic exposure among full-term and late preterm neonates is low (1.9%) [31].

### 4.3. Exclusion Criteria

Exclusion criteria included neonates aged ≥48 h at admission, those receiving antibiotic prophylaxis (for surgical intervention or congenital anomalies of the urinary tract), and cases with missing medical records or incomplete data.

### 4.4. Definitions

- Culture-proven sepsis or meningitis: defined as the isolation of a pathogen from blood and/or cerebrospinal fluid (CSF) cultures, or a positive result by polymerase chain reaction (PCR) [42,43].
- Culture-negative sepsis was defined as a clinical scenario in which an infant is evaluated for suspected sepsis, has negative bacterial cultures for pathogens, but exhibits clinical signs and/or supportive laboratory findings suggestive of sepsis, and is ultimately treated with a full course of empiric antibiotics (≥5 days) or until death based on clinical judgment [44,45].
- Contaminated blood cultures: isolation of bacteria that are commonly regarded as contaminants (e.g., coagulase-negative staphylococci [CoNS], *Micrococcus* species, or diphtheroids) from a single blood culture set, interpreted by clinicians as contamination rather than true bloodstream infection, and associated with discontinuation of antimicrobial therapy within 5 days [46].
- Pneumonia: a focal lower respiratory tract infection of the lung parenchyma, diagnosed by compatible clinical features and confirmed by positive local microbiological cultures and/or supportive imaging findings (e.g., chest X-ray, ultrasound), in the absence of concomitant bloodstream infection [47].
- Necrotizing enterocolitis (NEC): defined as Bell stage ≥ II [48], with compatible clinical signs and radiologic findings, including pneumatosis intestinalis, portal venous gas, or pneumoperitoneum.
- Short antibiotic course: administration of antibiotics for ≤48 h in neonates presenting with clinical signs of sepsis whose blood cultures are ultimately negative or deemed contaminated [32].
- Reinstitution of antibiotic treatment: a new course of antibiotics reinitiated 2–7 days after discontinuation of the previous course [32]. For each case, the indication for reinstitution (culture-proven sepsis, focal infection, culture-negative sepsis, or unknown reasons) was evaluated.
- EOS-related death: death occurring within the first 30 postnatal days.

### 4.5. Data Collection

Data were collected retrospectively by accessing the NICU computerized medical records (Metavision Suite, iMDSOFT, version 5.40.44, Tel Aviv, Israel). The following maternal and neonatal characteristics were evaluated: intrapartum antibiotic prophylaxis (IAP) administration, mode of delivery, group B *Streptococcus* antenatal screening, risk factors for EOS, gender, gestational age, birth weight, APGAR score at the 5th min, days on mechanical ventilation, blood and cerebrospinal fluid cultures, infecting organisms, sepsis (culture proven, culture-negative, focal infection and sepsis due to coagulase-negative staphylococci), mortality, and length of hospital stay. Data was obtained from computerized records by surveillance officers using a standardized form. 

### 4.6. Data Relating to Antibiotic Therapies

The following data were recorded: the timing of the initiation of the first antibiotic treatment and its duration; the drug used as the first course, any reinstitution and, finally, the overall duration of antibiotic therapies during hospitalization. The use of antibiotics was calculated as days of therapy (DOT): we assigned 1 DOT for each individual class of antibiotic administered during a single day. For example, two drugs in 1 day were calculated as 2 DOT [49].

### 4.7. Statistical Analyses

Analyses were performed using MedCalc® version 9.3 (MedCalc Software, Ostend, Belgium). For descriptive data comparisons, the Mann–Whitney test was used for non-parametric continuous data and the X2 test or Fisher exact test for categorical data. Non-parametric continuous variables are summarized as medians with quartiles (25th and 75th percentiles). Categorical variables are presented as percentages. The threshold for statistical significance was *p* < 0.05 for 2-sided tests.

## 5. Conclusions

In conclusion, our findings indicate an association between reduced use of CRP in the evaluation of suspected EOS and a decrease in laboratory investigations and antibiotic exposure among neonates. This strategy should be considered an integral component of neonatal antibiotic stewardship programs.

## Figures and Tables

**Table 1:** Demographics and clinical data of newborns. Calculation was performed excluding missing cases.

	All (*n* = 638)	Period 1 (*n* = 348)	Missing	Period 2 (*n* = 290)	Missing	*p*
Birth weight, wks, median, (IQR)	2555.0 (1650.0–3360.0)	2558.0 (1630.0–3387.5)	0	2555.0 (1690.0–3340.0)	0	1.0
Gestational age, wks, median, (IQR)	36.0 (32.0–39.0)	36.5 (32.0–39.0)	0	36.0 (32.0–39.0)	0	0.49
Male sex, *n*, (%)	362 (56.7)	205 (58.9)	0	157 (54.1)	0	0.26
IAP, *n*, (%)	381 (59.7)	217 (62.4)	7	164 (56.6)	1	0.16
Positive vagino-rectal screening swab, *n*, (%) *	82 (23.0)	47 (26.4)	170	35 (19.6)	111	0.16
Vaginal delivery, *n*, (%)	300 (47.0)	172 (49.4)	14	128 (44.1)	5	0.21
ROM > 18 h, *n*, (%)	106 (16.6)	48 (13.8)	54	58 (20.0)	15	0.18
Maternal fever during labor (>38 °C), *n*, (%)	17 (2.7)	3 (0.9)	21	14 (4.8)	2	<0.01
Mechanical ventilation, (%)	128 (20.1)	68 (19.5)	0	60 (20.7)	0	0.79
Culture proven sepsis (0–3 days), *n*, (%) §	9 (2.0)	5 (1.8)	0	4 (2.2)	0	0.91
Suspected sepsis (0–3 days), *n*, (%) §	16 (3.5)	11 (4.1)	0	5 (2.7)	0	0.60
Lumbar puncture (0–7 days), *n*, (%)	68 (10.7)	39 (11.2)	0	29 (10.0)	0	0.72
Meningitis (0–7 days), *n*, (%) †	5 (7.4)	4 (10.3)	0	1 (3.4)	0	0.56

IAP, intrapartum antibiotic prophylaxis; IQR, interquartile range; ROM, rupture of membranes. * The percentage was calculated among pregnant women who underwent prenatal screening. § The percentage was calculated among neonates who underwent blood culture. † The percentage was calculated among neonates who underwent lumbar puncture.

**Table 2:** Laboratory evaluations in period 1 and period 2.

	All (*n* = 638)	Period 1 (*n* = 348)	Missing	Period 2 (*n* = 290)	Missing	*p*
Blood culture obtained (0–3 days), *n* (%)	456 (71.5%)	271 (77.9%)	0	185 (63.8)	0	<0.001
CRP obtained (0–3 days), *n* (%)	258 (40.4%)	218 (62.6%)	0	40 (13.8%)	1	<0.001
>1 CRP (0–3 days) obtained, *n* (%)	51 (19.8)	48 (22.0)	0	3 (7.5)	1	0.06
CBC (0–3 days) obtained, *n* (%)	499 (78.2)	285 (81.9%)	0	214 (73.8)	1	0.02
>1 CBC obtained (0–3 days), *n* (%)	146 (29.3)	86 (30.2)	1	60 (28.0)	1	0.67
Lumbar puncture performed, *n* (%) ‡	55 (16.6)	33 (17.2)	0	22 (15.8)	0	0.86

CBC, complete blood count; CRP, C-reactive protein; ‡ Calculated on neonates with antibiotics initiated on day 0 to 3.

**Table 3:** Antibiotic treatments in period 1 and period 2 among all neonates and premature neonates under 34 weeks’ gestation.

All Neonates				
	All (*n* = 638)	Period 1 (*n* = 348)	Period 2 (*n* = 290)	*p*
Antibiotics initiated (0–3 days), *n* (%)	326 (51.1)	188 (54.0)	138 (47.6)	0.12
Short course, *n* (%) (≤48 h), *n* (%) *	186 (57.0)	98 (52.1)	88 (63.8)	<0.05
Duration of antibiotic treatment, median hours, (IQR) *	48.0 (36.0–64.0)	48.0 (37.0–72.0)	40.0 (35.0–60.0)	<0.001
DOT, median (IQR) *	4.0 (3.0–5.5)	4.0 (3.0–6.0)	3.5 (2.5–5.0)	<0.001
Antibiotics reinitiated, *n* (%) ‡	27 (8.2)	19 (10.0)	8 (5.8)	0.12
Neonates under 34 weeks’ gestation				
	All (*n* = 207)	Period 1 (*n* = 110)	Period 2 (*n* = 97)	*p*
Antibiotics initiated (0–3 days), *n* (%)	134 (64.7)	82 (74.5)	52 (53.6)	<0.01
Short course (≤48 h), *n* (%) §	113 (66.1)	43 (52.4)	41 (78.8)	<0.01
Duration of antibiotic treatment, median hours, (IQR) §	48.0 (36.0–60.0)	48.0 (44.3–60.0)	37.5 (36.0–48.0)	<0.001
DOT, median (IQR) §	4.0 (2.6–4.5)	4.0 (3.0–4.6)	3.2 (2.5–4.0)	<0.001

DOT, days of therapy. * Calculated on neonates with antibiotics initiated on day 0 to 3. Neonates with culture-proven infections (period 1, *n* = 5; period 2, *n* = 4) focal infection (period 1, *n* = 2; period 2, *n* = 0) and NEC (period 1, *n* = 0, period 2, *n* = 1) were excluded. ‡ Antibiotics reinitiated within 7 days from discontinuation (calculated on neonates with antibiotics initiated on day 0 to 3). § Calculated on neonates with antibiotics initiated on day 0 to 3. Neonates with culture-proven infections (period 1, *n* = 3; period 2, *n* = 1), necrotizing enterocolitis (period 1, *n* = 0; period 2, *n* = 1) or focal infection (period 1, *n* = 0; period 2, *n* = 0) were excluded.

## Data Availability

The raw data supporting the conclusion of this article will be made available by the authors on request.
